# Genome-Wide Identification of *BnaABI4* Genes and Their Roles in Regulating Stomatal Density and Drought Tolerance in *Brassica napus* L.

**DOI:** 10.3390/plants15121793

**Published:** 2026-06-10

**Authors:** Hui Yang, Minyu Tian, Jiban K. Kundu, Wenjing Deng, Yaqing Xiao, Chengfang Tan, Ying Ruan, Chunlin Liu

**Affiliations:** 1 Key Laboratory of Hunan Provincial on Crop Epigenetic Regulation and Development, College of Agronomy, Hunan Agricultural University, Changsha 410128, China; hncdhui_19@163.com (H.Y.); tianminyu1995@126.com (M.T.);; 2 Yuelushan Laboratory, Changsha 410128, China; 3 Plant Virus and Vector Interactions, Czech Agrifood Research Center (CARC), Drnovská 507, 16100 Prague, Czech Republic; jiban.kumar@carc.cz

**Keywords:** *Brassica napus* L., *BnaABI4*, stomatal density, drought tolerance, ABA signaling, transcriptome

## Abstract

Rapeseed (*Brassica napus* L.) growth and productivity are severely constrained by drought stress worldwide. Stomata are central regulators of plant transpiration and gas exchange, and therefore, represent key targets for enhancing water-use efficiency and drought tolerance. The transcription factor ABSCISIC ACID INSENSITIVE 4 (ABI4), a key regulator of the abscisic acid (ABA) signaling pathway, plays crucial roles in plant abiotic stress responses and stomatal regulation. Nevertheless, the biological functions of *BnaABI4* in *B. napus* remain largely unclear. In this study, four *BnaABI4* paralogs were identified in the elite rapeseed cultivar ZS11 through genome-wide identification and comprehensive bioinformatic analyses. Each BnaABI4 protein harbors only one conserved AP2 domain, and their promoters contain multiple stress/hormone-responsive cis-regulatory elements (CREs). We subsequently generated *BnaABI4-4* overexpression (OE) lines as well as *BnaABI4* CRISPR/Cas9-mediated knockout (KO) transgenic lines. Phenotypic assays demonstrated that OE line had reduced transpiration rate (Tr), stomatal conductance (Gs) and stomatal density, along with enhanced drought tolerance, whereas KO lines showed the opposite phenotype. Transcriptome profiling identified numerous differentially expressed genes (DEGs) enriched in biological pathways associated with stomatal regulation, ABA signal transduction, and drought acclimation. Further Gene Ontology (GO) and Kyoto Encyclopedia of Genes and Genomes (KEGG) enrichment analyses confirmed significant enrichment of DEGs in processes including stomatal development, stomatal movement, reactive oxygen species (ROS) homeostasis, and drought tolerance. Collectively, our findings demonstrate that *BnaABI4* negatively regulates stomatal density while positively contributing to drought tolerance in *B. napus*. This study lays a mechanistic foundation for genetic improvement and molecular breeding of drought-tolerant rapeseed cultivars.

## 1. Introduction

Rapeseed is the second most widely cultivated oilseed crop after soybean, and rapeseed oil ranks third among edible vegetable oils worldwide, occupying an indispensable position in safeguarding global oil supply security [1,2,3]. Beyond its primary role as an edible oil source, rapeseed possesses multiple economic and ecological values: it serves as a sustainable raw material for green biomass energy and chemical industries, with extensive applications in biodiesel production, industrial lubrication and anticorrosive coatings [4,5]. Additionally, its by-product, rapeseed meal, is an excellent resource for high-protein feed and organic fertilizer production [6]. Based on botanical morphological characteristics and agronomic traits, rapeseed is classified into four major cultivated species: *Brassica rapa* (AA genome), *Brassica napus* (AACC genome), *Brassica juncea* (AABB genome), and *Brassica carinata* (BBCC genome) [3,7,8]. Among them, *B. napus* stands out due to its superior agronomic traits, including high yield potential, high oil content, strong stress tolerance, and broad environmental adaptability, making it the most widely cultivated rapeseed species globally [3].

In recent decades, global climate warming has intensified dramatically, leading to an increased frequency of extreme weather events, particularly drought, which has become a major limiting factor for sustainable rapeseed production [9,10]. *B. napus* is highly sensitive to water deficit throughout the entire growth period, from seed germination to seed maturation, and even mild drought stress can cause significant physiological and metabolic disorders [11]. Specifically, drought stress at any developmental stage not only reduces seed oil content but also alters seed oil composition in rapeseed [12,13,14]. Furthermore, drought stress reduces leaf stomatal conductance (Gs) and transpiration rate (Tr), weakens photosynthetic carbon fixation efficiency, disturbs protein biosynthesis and secondary metabolite accumulation, inhibits plant growth, and ultimately results in pronounced reductions in seed yield and quality [15,16,17]. Therefore, there is a growing demand to improve drought tolerance in *B. napus* through biotechnology.

Numerous transcription factors (TFs) have been confirmed to regulate plant drought tolerance, including ABI4, NAC, CBF, bZIP, and MYB [18,19]. ABI4 belongs to a plant-specific TF family characterized by a single highly conserved AP2 domain at the N-terminus [20,21]. As a core regulatory component of the abscisic acid (ABA) signaling pathway, ABI4 plays vital roles in modulating multiple key biological processes in plants, including seed germination and dormancy, seedling root morphogenesis, stomatal development, and stomatal closure [22,23,24,25]. Increasing evidence indicates that ABI4 plays an essential role in plant responses to multiple abiotic stresses. For instance, the *Arabidopsis abi4* mutant fails to effectively induce the expression of *RBOHD* and subsequent ROS burst under ABA treatment, thereby impairing ABA-mediated stomatal closure and reducing drought resistance [25,26,27,28]. In contrast, the *abi4* mutant exhibits enhanced salt stress tolerance with shorter root length compared to the wild type (WT) under salt treatment, indicating that ABI4 negatively regulates salt tolerance in *Arabidopsis* [29,30].

The functional conservation and divergence of *ABI4* orthologs have been reported in various crop species. Overexpression of *OsABI4* significantly enhances drought tolerance in rice by regulating ABA signaling and stress-responsive gene expression [31]. Overexpression of *MtABI4* from *Medicago truncatula* in *Arabidopsis* enhances osmotic adjustment capacity and antioxidant levels, alleviates membrane oxidative damage, and ultimately improves cold stress tolerance [32]. Similarly, MdABI4 from apples positively regulates cold resistance, as evidenced by better growth of *MdABI4*-overexpressing apple calli and enhanced cold tolerance of *MdABI4*-overexpressing *Arabidopsis* and poplar seedlings compared to the WT [33]. In contrast, overexpression of peanut *AhABI4* in *Arabidopsis* aggravates growth inhibition and leaf wilting under salt stress, reducing salt tolerance [34], and downregulation of *TaABI4* in wheat weakens ABA sensitivity and reduces ROS accumulation, thereby improving salt tolerance [35]. Furthermore, maize ZmABI4 can directly bind to the promoters of drought-responsive genes *Zmrab17* and *ZmADH1* to activate their transcription, and it can rescue the ABA-insensitive phenotype of the *Arabidopsis abi4* mutant, suggesting functional conservation of *ZmABI4* in plant drought adaptation [36]. Collectively, these findings indicate that ABI4 plays diverse roles in regulating plant abiotic stress responses including drought stress, but its function in *B. napus* remains unclear.

Stomata, specialized epidermal structures formed by two guard cells, are critical gateways for water transpiration and gas exchange between plants and the external environment [37,38]. Plants can reduce transpirational water loss and adapt to drought stress by remodeling stomatal structure and modulating stomatal closure [39]. Previous studies have demonstrated that TFs can modulate water loss and drought tolerance by regulating stomatal density and stomatal closure. In *Arabidopsis*, ABI4 [25,28], NAC019 [40], and NAC055 [41] positively regulate stomatal closure and drought tolerance, whereas AGL16 negatively modulates drought resistance by increasing stomatal density and repressing stomatal closure [42]; in maize, ZmNAC49 reduces stomatal density to improve drought tolerance [43], and ZmPIF1 enhances drought tolerance by inducing stomatal closure [44]; in potato, StWRKY46 promotes stomatal closure and thereby enhances drought tolerance [45]; in sorghum, SbMYC2 reduces stomatal density and promotes drought adaptation [46]. Additionally, in Shanxin poplar, PdbMYB44 confers drought tolerance by reducing stomatal aperture [47].

Nevertheless, the regulatory role of ABI4 in stomatal density modulation, as well as the biological functions of BnaABI4 in *B. napus* remain largely uncharacterized to date. In this study, we first performed a systematic bioinformatic analysis of BnaABI4, and further generated *BnaABI4-4* overexpression transgenic lines (OE) and four *BnaABI4* gene-edited knockout lines (KO-1, KO-2, KO-3, KO-4). Phenotypic characterization combined with transcriptome RNA-seq analysis revealed that BnaABI4 positively regulates plant drought tolerance by modulating stomatal density, mediating stomatal closure, and coordinating the expression of drought-responsive genes. This study provides novel insights into the molecular mechanisms underlying *BnaABI4*-mediated drought tolerance and stomatal density regulation and clarifies the regulatory role of *ABI4* in modulating stomatal density. Meanwhile, it lays a solid foundation for the genetic improvement and molecular breeding of drought-tolerant *B. napus* varieties.

## 2. Results

### 2.1. Identification and Phylogenetic Analysis of BnaABI4 Genes in B. napus

Genome-wide identification of *BnaABI4* genes in *B. napus* cv. ZS11 was carried out using the BnIR database. A total of four members of *BnaABI4* (*BnaABI4-1*, *BnaABI4-2*, *BnaABI4-3*, *BnaABI4-4*) paralogs were identified. The detailed physicochemical characteristics of these genes and their encoded proteins, including gene ID, chromosomal location, isoelectric point, molecular weight, GRAVY value, and protein length, are summarized in Table S1.

**Figure 1:**
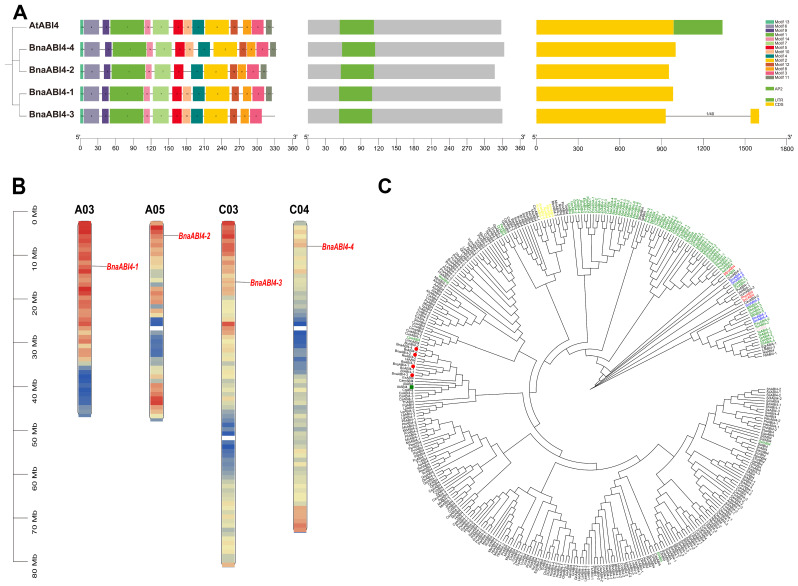
Characterization analysis of *BnaABI4* genes in *B. napus*. (**A**) Conserved motifs, conserved domains, and gene structures of BnaABI4 proteins. For better visualization, the intron of *BnaABI4-3* was scaled to 1/40 its original length. (**B**) Chromosomal localization of the *BnaABI4* genes in *B. napus.* (**C**) Phylogenetic tree of ABI4 proteins in *B. napus* and other plants. A total of 368 ABI4 protein sequences derived from 239 plant species were used for phylogenetic reconstruction. BnaABI4 proteins were marked with red circles and AtABI4 with green square. Bryophytes, pteridophytes, dicotyledons, monocotyledons and gymnosperms are labeled in red, blue, black, green and magenta, respectively, while basal angiosperms are indicated in yellow.

BnaABI4 proteins belong to the AP2/ERF superfamily and possess a typical AP2 conserved domain (Figure 1A). Gene structure analysis showed that most *BnaABI4* genes are intron-free, with only *BnaABI4-3* harboring one intron (Figure 1A). Conserved motif analysis revealed that the overall motif distribution of BnaABI4 proteins was identical to AtABI4, except that BnaABI4-2 lacked motif 10 and BnaABI4-3 lacked motif 11 (Figure 1A). The four *BnaABI4* genes were situated on distinct chromosomes: *BnaABI4-1* on chromosome A3, *BnaABI4-2* on chromosome A5, *BnaABI4-3* on chromosome C3 and *BnaABI4-4* on chromosome C4 (Figure 1B). In this study, a total of 368 ABI4 amino acid sequences from *B. napus* and 238 other plant species, encompassing bryophytes, pteridophytes, gymnosperms, and angiosperms, were used to construct the phylogenetic tree (Figure 1C). Phylogenetic analysis revealed that ABI4 proteins of all *Brassicaceae* species, including the BnaABI4 members, clustered within a single subclade.

### 2.2. Homology Analysis of BnaABI4 Proteins

**Figure 2:**
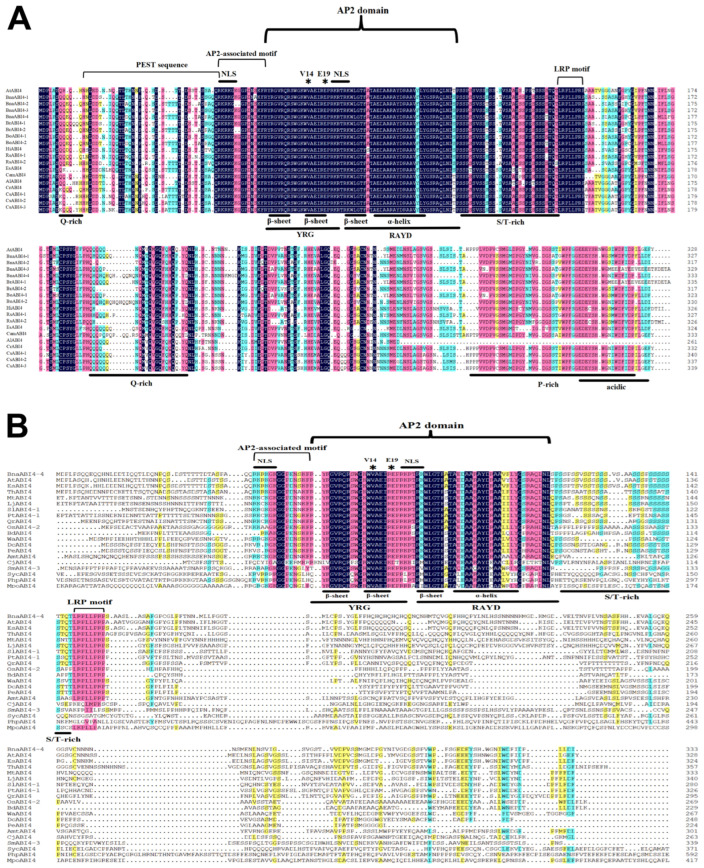
(**A**) Homology comparison of ABI4 amino acid sequences within the *Brassicaceae* family. (**B**) The amino acid sequences of BnaABI4 from *B. napus* were analyzed in comparison to those from 19 other plant species. * indicates the amino acid residues at positions 14 (V14) and 19 (E19), respectively. The black background, red background, blue background, and the yellow background represent the similarity ratio gradient of amino acid sequences among several species, which is 100%, greater than or equal to 70%, greater than or equal to 50%, and greater than or equal to 33%, respectively.

To explore the ABI4 sequence conservation within the *Brassicaceae* family, multiple sequence alignment was conducted on ABI4 amino acid sequences from all *Brassicaceae* species clustered on the same phylogenetic branch. As shown in Figure 2A, the overall sequence similarity of the 19 *Brassicaceae* ABI4 proteins reached 76.15%. These results indicate that ABI4 proteins are highly evolutionarily conserved across *Brassicaceae* species, exhibiting relatively low sequence divergence, suggesting that their biological functions are also relatively conserved. The AP2 domain, composed of 59 amino acids, exhibited strong conservation among all *Brassicaceae* ABI4 sequences, with amino acid variations detected only at three sites (positions 32, 47, and 58).

To investigate the sequence conservation across different families, multiple sequence alignment was performed using ABI4 amino acid sequences from *B. napus* and another 19 additional representative plant species (Figure 2B). These representative species belong to 15 plant families, mainly covering model species of each family, *Tarenaya hassleriana* (ThABI4) from *Cleomaceae* which is evolutionarily closely related to *B. napus* (BnaABI4), and *Syntrichia caninervis* (SycABI4), a pivotal species of *Pottiaceae* with great significance for the study of plant evolutionary origins. As shown in Figure 2B, the overall sequence similarity among the 20 ABI4 proteins was only 19.72%, whereas the AP2 domain and its immediately flanking N-terminal and C-terminal regions were highly conserved. Collectively, these results revealed that ABI4 proteins share low overall sequence homology across different plant families. Although the full-length ABI4 amino acid sequences have diverged substantially during plant evolution, the AP2 conserved domain and core functional regions maintain a high degree of sequence conservation.

### 2.3. Promoter Cis-Regulatory Elements Analysis

**Figure 3:**
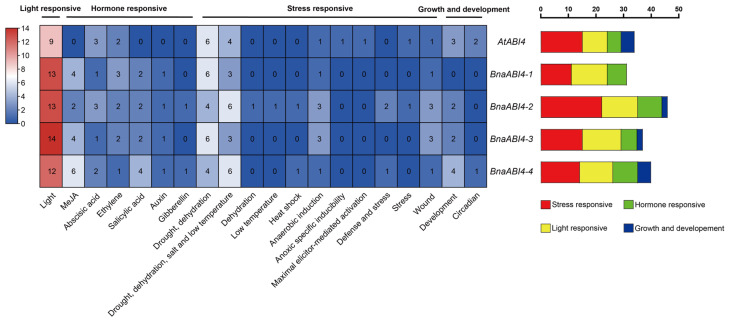
Analysis of CREs in the upstream promoter regions of *BnaABI4* genes. The number represents the number of CREs. Different categories are shown with different colour blocks.

The promoter regions of *BnaABI4* and *AtABI4* genes were analyzed using the PlantCARE database to identify cis-regulatory elements (CREs). This analysis facilitated inference of the potential transcriptional regulatory characteristics and expression control mechanisms of *BnaABI4* genes. The identified CREs were classified into four categories: stress-responsive elements, light-responsive elements, hormone-responsive elements, and plant growth and development elements (Figure 3). Collectively, these results indicate that *BnaABI4* genes may be involved in multiple biological processes, including stress responses, hormone signal transduction, and plant growth and development.

The composition of CREs in the promoters of *BnaABI4* genes is similar to that of *AtABI4* from the model plant *Arabidopsis thaliana*, while also exhibiting distinct differences (Figure 3). This suggests that *BnaABI4* genes have retained functional conservation during evolution, while acquiring species-specific divergent features. Furthermore, the four *BnaABI4* paralogs showed both similarities and differences in their promoter CRE profiles, implying that these four *BnaABI4* paralogs not only possess functional coordination but also have undergone distinct functional differentiation and specialization.

### 2.4. Subcellular Localization of BnaABI4 Genes

**Figure 4:**
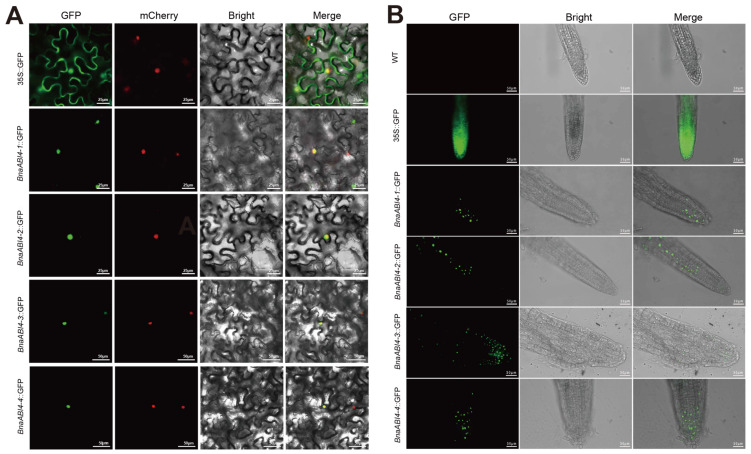
Subcellular localization analysis of BnaABI4 proteins. (**A**) Subcellular localization of BnaABI4-GFP fusion proteins transiently expressed in tobacco leaf epidermal cells. Images show GFP fluorescence, mCherry fluorescence, bright-field and merged images. (**B**) Subcellular localization of BnaABI4-GFP fusion proteins heterologously expressed in young roots of *Arabidopsis thaliana*. Images show GFP fluorescence, bright-field and merged images.

According to subcellular localisation prediction by WoLF-PSORT, all the four BnaABI4 proteins localise to the nucleus (Table S1), which is consistent with their role as transcriptional regulators. To validate this prediction, we constructed and transiently expressed the BnaABI4-GFP fusion constructs in *Nicotiana benthamiana* leaves, with pBI121-NLS-mCherry serving as the nucleus marker and the empty p27-GFP vector serving as the control. As shown in Figure 4A, the GFP signal from the empty vector was diffusely distributed throughout the cells, whereas the fluorescence signal of all the four BnaABI4-GFP were detected in the nucleus. These results indicate that BnaABI4 proteins were localized in the nucleus, suggesting its potential involvement in nucleus-associated signal transduction and nuclear regulatory processes.

Meanwhile, BnaABI4-GFP fusion expression vectors were heterologously transformed into *Arabidopsis thaliana*. As shown in Figure 4B, strong GFP fluorescence was specifically enriched in the nucleus of root tip cells from stably transformed *Arabidopsis* lines. This confirms that all four BnaABI4 proteins are typical nuclear-localized proteins. Collectively, these results indicate that the nuclear localization pattern of BnaABI4 paralogs exhibits genetic stability and evolutionary conservation, rather than representing an artifact of transient expression systems, further verifying the reliability and authenticity of the subcellular localization results.

In summary, all the four BnaABI4 proteins were nuclear localization proteins, suggesting that it mainly played its biological function by binding DNA in the nucleus and regulating the expression of downstream genes.

### 2.5. Overexpression of BnaABI4-4 Reduces Stomatal Density, While BnaABI4 Knockout Mutants Increase Stomatal Density

**Figure 5:**
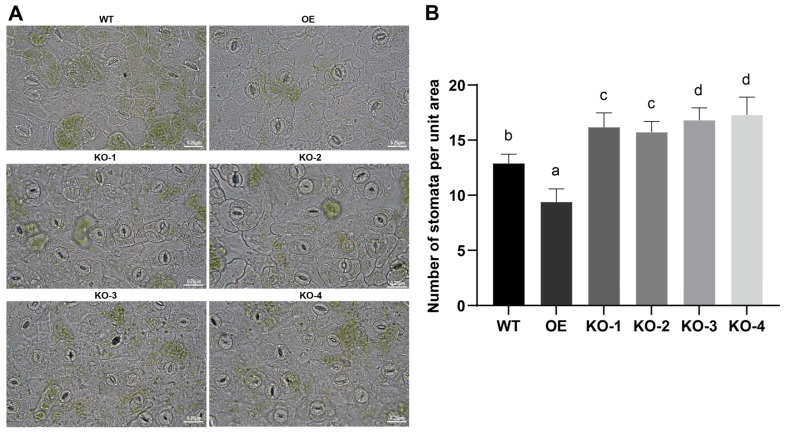
Stomatal density of ZS11 and *BnaABI4* transgenic lines. (**A**) Stomatal phenotypes of WT and transgenic plants. Scale bar = 0.25 µm. (**B**) Number of stomata per unit area. Each value represents the average ± SD of thirty independent biological replicates. Same lowercase letters denote no significant difference (*p* > 0.05), and different lowercase letters indicate significant differences (*p* < 0.05).

The stomatal density of wild-type ZS11 (WT), *BnaABI4-4* overexpressing (OE) and *BnaABI4* knockout (KO) rapeseed plants was observed under a Leica microscope. Four KO lines were generated in this study: KO-2 with simultaneous knockout of both *BnaABI4-1* and *BnaABI4-2*; KO-1 with simultaneous knockout of *BnaABI4-1*, *BnaABI4-2*, and *BnaABI4-3*; KO-3 with simultaneous knockout of *BnaABI4-1*, *BnaABI4-2*, and *BnaABI4-4*; and KO-4 with knockout of all four *BnaABI4* paralogs. It was found that the stomatal density of OE plants was significantly lower than that of WT, whereas the stomatal density of KO plants was significantly greater than that of WT (Figure 5A). Specifically, the stomatal density of OE plants was 0.73 times that of WT, whereas that of KO-1, KO-2, KO-3 and KO-4 plants was 1.26, 1.22, 1.30, and 1.34 times that of WT, respectively (Figure 5B) (Table S2). Collectively, these results indicate that *BnaABI4* negatively regulates stomatal density in *B. napus*, and *BnaABI4* paralogs play a synergistic regulatory role in stomatal density.

### 2.6. BnaABI4 Positively Regulates Drought Tolerance in B. napus

**Figure 6:**
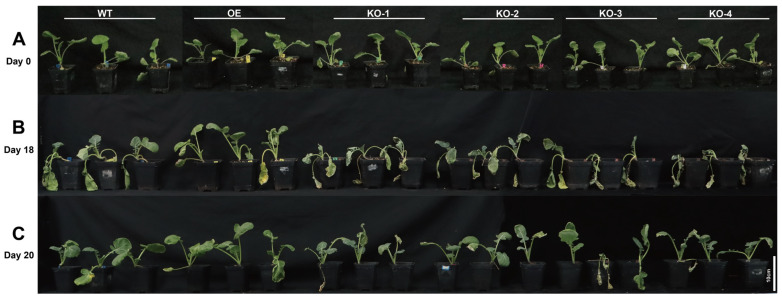
Phenotypic characteristics of *B. napus* plants with different genotypes before drought treatment, under drought stress, and after rewatering. (**A**) Before drought treatment; (**B**) At the stage with obvious phenotypic symptoms under drought stress; (**C**) 2 days after rewatering. WT, wild-type ZS11; OE, *BnaABI4-4*-overexpressing lines; KO-1–KO-4, *BnaABI4* knockout lines; Scale bar = 10 cm.

Under drought stress, all genotypes of *B. napus* exhibited typical drought-related phenotypes including wilting and chlorosis, with significant differences in the onset time, severity of phenotypic symptoms, and recovery capacity after rewatering (Figure 6). The OE plants displayed enhanced drought tolerance with milder wilting symptoms and a faster recovery capacity following rewatering. In contrast, all KO lines were more sensitive to drought stress, showing severe wilting and weaker recovery ability after rewatering. Among all KO lines, the double-gene knockout line KO-2 possessed higher drought tolerance than the triple- or quadruple-gene simultaneously edited lines (KO-1, KO-3, KO-4). The phenotypic performance and recovery capacity of wild-type ZS11 were intermediate between the OE and KO lines. These results indicate that *BnaABI4* positively regulates drought tolerance in *B. napus*, and *BnaABI4* paralogs play a synergistic regulatory role in the drought stress response of *B. napus*.

### 2.7. BnaABI4 Genes Decrease Tr and Gs

**Figure 7:**
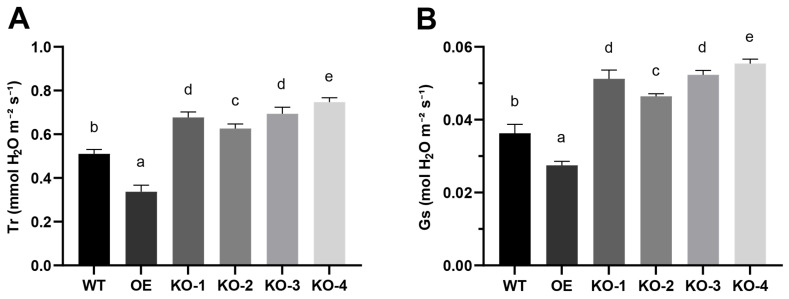
Effects of *BnaABI4* expression on leaf water and gas exchange in *B. napus*. All measurements were performed on the third fully expanded leaf from the top. (**A**) Tr, (**B**) Gs. Each value represents the average ± SD of three independent biological replicates. Same lowercase letters denote no significant difference (*p* > 0.05), and different lowercase letters indicate significant differences (*p* < 0.05).

To further explore the physiological mechanisms of *BnaABI4* genes in response to drought stress, we measured Tr and Gs in the third fully expanded leaf from the top of plants under drought conditions (Figure 7A,B). After drought treatment, the Tr and Gs of OE line decreased by 33.98% and 24.24%, respectively, compared with WT. Conversely, KO lines displayed the opposite phenotypes. The Tr of KO-1 to KO-4 increased by 32.69%, 22.88%, 35.94% and 46.41%, while their Gs increased by 14.10%, 12.78%, 14.41% and 15.26%, relative to WT.

### 2.8. Transcriptomic Analysis of BnaABI4-4-Overexpressing Lines

#### 2.8.1. Global Expression Profiling

**Figure 8:**
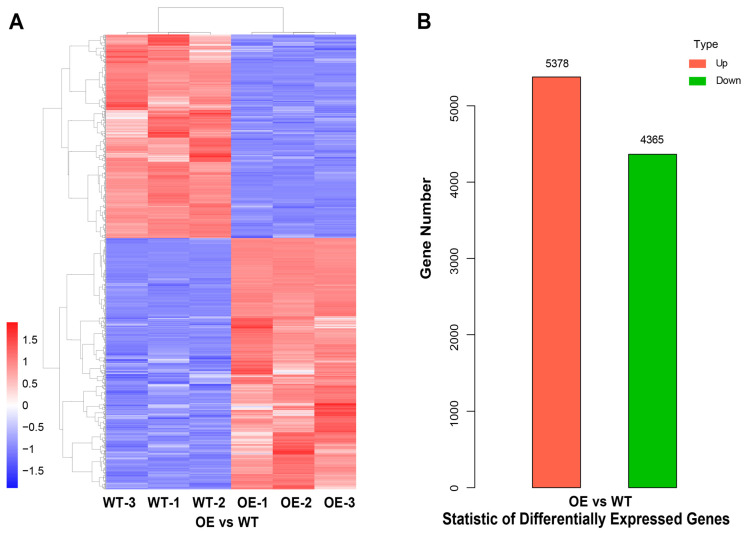
Statistics and clustering analysis of DEGs between OE and WT of *B. napus*. (**A**) Cluster analysis results of different groups; (**B**) Statistic of different expressed genes.

Transcriptome analysis results are presented in the Supplementary Materials. A total of more than 45 Gb of raw data were generated from the RNA-seq libraries (Table S3). For all samples, the Q20 ratio, Q30 ratio, and GC content were higher than 99%, 98%, and 47%, respectively. The effective rate of each sample was over 97%, and more than 97% of the clean reads were successfully mapped to the reference genome of *B. napus* cv. ZS11. Using the screening thresholds of ∣log_2_FC∣ > 1 and *p* < 0.05, a total of 9743 significantly differentially expressed genes (DEGs) were identified (Figure 8A), including 5378 upregulated genes and 4365 downregulated genes (Figure 8B).

#### 2.8.2. Functional Enrichment Analysis

**Figure 9:**
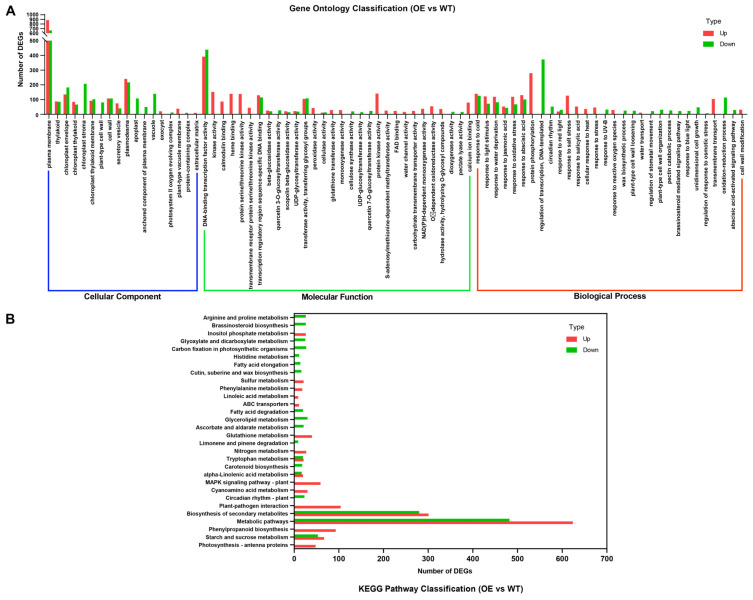
GO and KEGG Analyses of the DEGs OE vs. WT in *B. napus*. (**A**) GO classification of the DEGs; (**B**) KEGG classification of the DEGs.

GO analysis was performed to characterize the functional distribution of DEGs (Figure 9A). In the cellular component (CC) category, DEGs were mainly enriched in plasma membrane- and chloroplast-related compartments, as well as cell wall- and vesicle-associated structures, which are closely related to photosynthesis, signal perception, material transport and cell wall remodeling, supporting stomatal development and stress adaptation. For molecular function (MF), enriched terms covered transcription factor activity, kinase-mediated phosphorylation, calcium-dependent signaling, glycosyltransferase/hydrolase activities, antioxidant functions and transmembrane transport activities. These functions contribute to transcriptional regulation, signal transduction, cell wall metabolism, redox balance and substance transport, underlying stomatal formation and drought tolerance. In biological process (BP), DEGs were enriched in phytohormone and multiple stress responses, light-associated pathways and circadian rhythm. Key processes linked to drought tolerance and stomatal traits were highlighted, including stomatal movement regulation, wax biosynthesis, cell wall modification, water transport and osmotic stress response. Hormone signaling, phosphorylation, redox reactions and transcriptional regulation were also enriched. Overall, DEGs respond to drought stress via coordination of light signaling, circadian rhythm, phytohormone pathways, redox homeostasis, cell wall remodeling and stomatal regulation.

KEGG enrichment analysis revealed that the DEGs were remarkably enriched in photosynthesis-antenna proteins, circadian rhythm, cutin, suberine and wax biosynthesi, phenylpropanoid biosynthesis, MAPK signaling pathway, starch and sucrose metabolism, and carotenoid biosynthesis (Figure 9B). These pathways cooperatively regulate stomatal development, stomatal movement, ROS homeostasis and drought adaptation, indicating that the candidate gene mediates plant drought tolerance by coordinating multiple physiological and molecular processes.

#### 2.8.3. BnaABI4 Modulates Genes Controlling Stomatal Traits and Drought Tolerance

Stomatal density and development are key morphological determinants of plant drought tolerance by regulating leaf water loss [48,49]. Transcriptome analysis revealed numerous DEGs related to stomatal development. Positive regulators of stomatal density (*BnaSPCH*, *BnaSCRM*, *BnaSCRM2*) were significantly downregulated in OE lines [50,51], whereas the negative regulator *BnaMYC2* was upregulated [46]. As a drought-inducible transcription factor, elevated *BnaMYC2* further activates downstream drought-responsive and ROS-scavenging genes, coordinating stomatal development and stress adaptation [46,52].

Stomatal closure is a rapid drought-adaptive strategy. Multiple guard-cell-related genes governing stomatal closure were differentially expressed in OE plants. Upregulated *BnaCNGC12*, *BnaBON3*, *BnaRBOHD* and *BnaRBOHF* collectively promote ABA-dependent Ca^2+^ signaling, ROS generation and ion channel activation to trigger stomatal closure [53,54,55,56]. The ROS homeostasis-related gene *BnaGSTU17* was downregulated, facilitating ABA accumulation and stomatal closure [57,58]. Antioxidant genes *BnaMDAR2* and *BnaALDH3I1* were induced to scavenge excess ROS, relieve oxidative damage and stabilize guard cell function [59,60]. In addition, exocyst complex-encoding *BnaEXO70B2* was upregulated to mediate ABA-independent stomatal closure via vesicle trafficking and membrane remodeling, cooperating with canonical ABA signaling to maintain leaf water homeostasis [61,62].

Genes involved in core drought-resistance pathways were also differentially expressed to form an integrated drought defense network with stomatal regulators. ABA biosynthetic gene *BnaNCED9* and *BnaABI4-4* were upregulated to enhance ABA accumulation [25,28,63,64]. ABA signaling cascade components *BnaRAF43* and *BnaSnRK2.2* were induced to amplify drought signaling [65,66,67]. By contrast, ABA signaling repressors (*BnaABR*, *BnaCHR12*, *BnaOCP3*) and ABA catabolic genes (*BnaCYP707A1/2/4*) were downregulated, reinforcing ABA-mediated drought responses [68,69,70,71,72,73]. For osmotic and membrane stability regulation, LEA family genes (*BnaLEA3*, *BnaLEA18*) and phosphatidylcholine synthesis-related genes (*BnaNMT2*, *BnaNMT3*) were upregulated to mitigate cellular dehydration and stabilize plasma membranes [74,75,76,77]. Cell wall-remodeling genes were also altered: the negative regulator *BnaXTH6* was downregulated while the positive regulator *BnaGALT3* was upregulated, strengthening epidermal cell walls and reducing water loss [78,79].

Multiple drought-responsive transcription factors, including *BnaNAC019*, *BnaNAC055*, *BnaWRKY33*, *BnaDREB2A*, *BnaDREB2B* and *BnaDDF1*, were significantly upregulated in OE lines. These transcription factors cooperate with *BnaABI4-4* to activate downstream stomatal- and drought-related genes, improve osmotic tolerance and maintain ROS homeostasis [20,40,41,80,81,82,83,84,85].

Notably, drought marker genes *BnaRD29A/B* were downregulated under normal conditions in OE plants, indicating a “low baseline, strong induction” drought-adaptation pattern consistent with previous *Arabidopsis* drought-resistant mutants [86,87]. Low basal expression of stress-related genes avoids unnecessary growth energy consumption, whereas rapid activation under drought stress achieves a balance between plant growth and drought resistance.

#### 2.8.4. Validation of RNA-Seq Data by RT-qPCR

**Figure 10:**
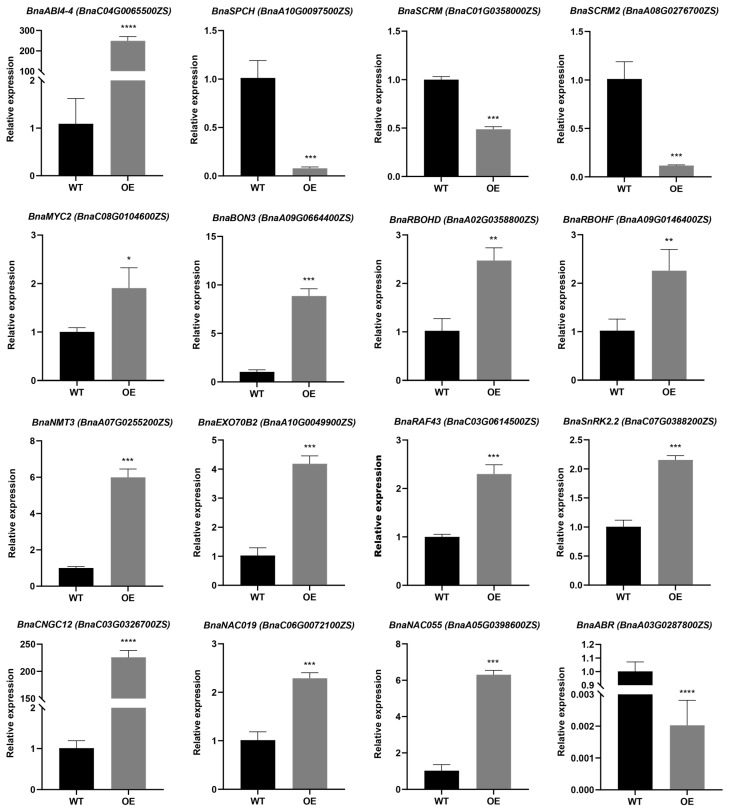
Validation of RNA-seq data by RT-qPCR. Each value represents the average ± SD of three independent biological replicates. Asterisks indicate significant differences: * *p* < 0.05, ** *p* < 0.01, *** *p* < 0.001, **** *p* < 0.0001.

To verify the reliability of RNA-seq expression data, *BnaABI4-4* and 15 candidate genes involved in stomatal traits and drought tolerance were selected for RT-qPCR analysis. The expression trends determined by RT-qPCR were highly consistent with those from RNA-seq, confirming the accuracy of transcriptome data (Figure 10).

## 3. Discussion

Drought represents a major constraint limiting the productivity of *B. napus*, and elucidating the molecular mechanisms underlying drought tolerance is essential for the genetic improvement of this important rapeseed crop. As a core component of the ABA signaling pathway, ABI4 has been well documented to be involved in abiotic stress responses across various plant species; however, its functional characterization in *B. napus* has remained elusive. To address this research gap, the present study systematically identified four *BnaABI4* paralogs (*BnaABI4-1* to *BnaABI4-4*) in *B. napus* cv. ZS11 and comprehensively characterized their roles in stomatal density regulation and drought tolerance, thereby extending our understanding of *ABI4*-mediated stress adaptation in polyploid oilseed crops. Consistent with findings from previous studies on *ABI4* orthologs in *Arabidopsis*, rice, and apple [25,31,33], our results confirm that BnaABI4 functions as a positive regulator of drought tolerance, reflecting the functional conservation of ABI4 in plant drought responses. Notably, compared with other plant species, the specific regulatory pathway of BnaABI4 in *B. napus* exhibits distinct features, particularly in the modulation of stomatal density and the coordination of multiple signaling pathways.

Bioinformatic analyses revealed that BnaABI4 proteins belong to the AP2/ERF transcription factor superfamily, harboring a highly conserved AP2 domain with high sequence conservation across *Brassicaceae* species. This is consistent with the notion that the core functions of ABI4 are evolutionarily conserved in plants [20,21]. The four *BnaABI4* genes are respectively localized to chromosomes A3, A5, C3 and C4, which is in accordance with the allopolyploid (AACC) genomic origin of *B. napus*. This indicates that *BnaABI4* paralogs were inherited from its diploid progenitors (*B. rapa* and *B. oleracea*) and may have undergone functional divergence during polyploidization [7,88]. Numerous CREs responsive to ABA, drought and other stresses, as well as phytohormones, are identified in the promoter regions of *BnaABI4* genes, suggesting that these genes integrate multiple environmental and endogenous signals to modulate plant growth, development and stress responses. This inference is further supported by transcriptomic data showing that DEGs are significantly enriched in GO terms associated with phytohormone response and stress adaptation. Collectively, these findings validate the evolutionary conservation of ABI4 and imply that BnaABI4 has evolved species-specific regulatory patterns to accommodate the unique developmental and stress-adaptive requirements of *B. napus*.

Phylogenetic analysis of ABI4 protein sequences from 239 plant species further verifies that ABI4 is a plant-specific transcription factor broadly distributed in major plant groups [23]. Its occurrence in bryophytes and pteridophytes suggests an origin during plant terrestrialization, supporting its vital roles in land-plant adaptation [21]. ABI4 displays conserved vertical evolution with adaptive divergence; this balance between conservation and variation maintains core function stability while enabling evolutionary flexibility for species-specific adaptation.

A key novel finding of the present study is that BnaABI4 negatively regulates stomatal density in *B. napus*. Previous studies have demonstrated that *Arabidopsis* ABI4 functions in mediating stomatal closure [25,28], yet its regulatory role in stomatal density has not been documented to date. Phenotypic analyses herein revealed that stomatal density in OE lines was 0.73-fold that in WT, whereas KO displayed a 1.22–1.34-fold higher stomatal density compared with WT, among which the quadruple mutant exhibited the maximum stomatal density. Further transcriptomic analysis indicated that BnaABI4 modulates stomatal density via repressing positive regulatory genes of stomatal development (*BnaSPCH*, *BnaSCRM*, *BnaSCRM2*) and activating the negative regulator *BnaMYC2* [46,50,51]. Such a regulatory module is consistent with other transcription factors conferring drought tolerance through modulating stomatal density, including *SbMYC2* and *ZmNAC49* [43,46]. Stomatal density is a key structural factor determining leaf gas exchange efficiency and water transpiration in plants [37,38]. Our results revealed that under drought stress, overexpression of *BnaABI4-4* markedly reduced leaf Tr and Gs, while *BnaABI4* knockout significantly increased these two physiological indices. These findings confirm that BnaABI4 modulates stomatal density to reshape leaf stomatal conduction properties, reduces drought-induced water loss, and thereby positively regulates drought response in rapeseed. Collectively, this study expands the known functional spectrum of ABI4 and unravels the unique function of BnaABI4 in governing stomatal development in *B. napus*.

The present study verifies that BnaABI4 is functionally conserved in drought responses and shares analogous functions with ABI4 orthologs in other crop species. Overexpression of *OsABI4* in rice [31] and *MdABI4* in apple [33] has been demonstrated to enhance plant drought tolerance. Consistently, OE lines show reduced stomatal density and improved drought tolerance, whereas KO lines exhibit the opposite phenotype. Notably, the double-gene knockout line KO-2 displays greater drought tolerance than triple- and quadruple-gene knockout lines, suggesting that *BnaABI4* paralogs exert synergistic regulation during drought responses, and excessive knockout of *BnaABI4* members may disrupt the homeostatic balance of stress-signaling pathways. This observation aligns with the evolutionary features of functional redundancy and divergence among homologous genes in polyploid plants, which constitute a widespread adaptive strategy for plants to cope with fluctuating environments [3,88]. Transcriptomic data further reveal that the drought-tolerant function of BnaABI4 is tightly linked to its modulation of stomatal traits and the ABA signaling pathway.

Transcriptomic analysis further unravel the molecular mechanism underlying *BnaABI4*-mediated drought tolerance. DEGs between OE lines and WT in *B. napus* are significantly enriched in multiple pathways associated with drought adaptation, including stomatal development, stomatal movement, ABA signaling, ROS homeostasis, cell wall remodeling and cuticular wax biosynthesis. Specifically, BnaABI4 reinforces the ABA signaling cascade by upregulating ABA biosynthetic genes (*BnaNCED9*) [63,64] and signaling-related genes (*BnaRAF43*, *BnaSnRK2.2*) [65,66,67], while downregulating ABA catabolic genes (*BnaCYP707A1/2/4*) [72,73] and negative regulators (*BnaABR*, *BnaCHR12*) [68,69,70], thereby promoting stomatal closure and drought responses. Meanwhile, BnaABI4 modulates ROS homeostasis and alleviates drought-induced oxidative damage via upregulating antioxidant-encoding genes (*BnaMDAR2*, *BnaALDH3I1*) [59,60] and repressing *BnaGSTU17* [57,58]. In addition, elevated expression of *BnaEXO70B2* suggests that BnaABI4 mediates ABA-independent stomatal closure through vesicular trafficking [61,62], which cooperates with the canonical ABA signaling pathway to maintain plant water homeostasis. Collectively, these findings demonstrate that BnaABI4 confers drought tolerance via an integrated multi-pathway regulatory network, validating our core hypothesis that BnaABI4 enhances drought adaptability by orchestrating multiple physiological and molecular processes.

Notably, under normal growth conditions, the expression of canonical drought-marker genes *BnaRD29A/B* is downregulated in OE lines, exhibiting a regulatory pattern characterized by low basal expression and strong stress-induced induction. This pattern is consistent with that observed in *Arabidopsis* OE-*CBF2* plants [86] and drought-tolerant *WRKY* quintuple mutants [87]. Such a regulatory strategy avoids growth retardation caused by constitutive high expression of stress-responsive genes under non-stress conditions, thereby balancing plant growth and drought tolerance. Given that *B. napus* demands a trade-off between high yield and drought tolerance [1,3], this regulatory module possesses great practical significance: overexpressing *BnaABI4* can enhance drought tolerance without compromising normal plant growth, laying a solid foundation for its application in molecular breeding.

The scientific significance of the present study is mainly reflected in four aspects. First, we systematically identified and functionally characterized the *BnaABI4* genes in *B. napus*, filling the research gap of ABI4-related investigations in this important oilseed crop. Second, we uncovered a novel function of ABI4 in regulating stomatal density, broadening the known functional scope of the ABI4 transcription factor. Third, we elucidated the molecular mechanism by which BnaABI4 confers drought tolerance via synergistically modulating stomatal development, ABA signaling and ROS homeostasis. Fourth, we validated the synergistic roles of *BnaABI4* paralogs in drought responses, providing new insights into the functional evolution of homologs in polyploid plants. Collectively, these findings not only deepen our theoretical understanding of plant drought adaptation mechanisms but also provide elite candidate genes for the genetic improvement of drought-tolerant *B. napus* varieties.

Despite the advances achieved in the present study, several limitations remain, which also highlight directions for future research. First, specific interacting partners of the BnaABI4 protein need to be identified to unravel the fine-tuned molecular network governing its regulation of stomatal development and drought responses. Second, the four *BnaABI4* paralogs exhibit differences in promoter CREs and expression patterns, and their functional divergence requires further validation. Third, future studies should explore the roles of BnaABI4 under other abiotic stresses, such as salinity and cold stress, as well as its crosstalk mechanisms with other phytohormone signaling pathways including jasmonic acid (JA) and salicylic acid (SA). Further investigations along these lines will deepen our comprehensive understanding of BnaABI4 functions and provide additional candidate gene resources for the genetic improvement of drought-tolerant *B. napus* varieties.

## 4. Materials and Methods

### 4.1. Bioinformatics Analysis

The genome data for *B. napus* cv. ZS11 were downloaded from the BnIR database (https://yanglab.hzau.edu.cn/BnIR, 5 March 2023) [89]. To identify *BnaABI4* genes, *AtABI4* (*AT2G40220*) [90] was used as the query for gene searches against the *B. napus* cv. ZS11.v0 dataset.

Physicochemical properties of BnaABI4 proteins were analyzed using the ProtParam tool from the ExPASy website (https://web.expasy.org/protparam/, 15 October 2024) [91]. MEME Suite v.5.5.9 was used to investigate the conserved motifs of BnaABI4 proteins [92]. The NCBI Conserved Domain Database (CDD) was utilized to predict and annotate conserved functional domains [93]. The conserved motifs, conserved domains and gene structure of BnaABI4 proteins were visualized using the BioSequence Structure Illustrator module in TBtools. Furthermore, the genomic location information of *BnaABI4* genes was extracted from the *B. napus* cv. ZS11.v0 genome assembly. Chromosomal distribution of these genes was mapped via the Show Genes on Chromosome function in TBtools [94].

For phylogenetic analysis, the amino acid sequences of BnaABI4 were retrieved from NCBI [95], Phytozome [96], and EnsemblPlants database [97] (Table S4). All sequences were aligned using ClustalW implemented in MEGA v.11.0.13 [98]. The phylogenetic tree was performed using the neighbor-joining (NJ) method with 1000 bootstrap replicates, based on the Poisson correction model and visualized via Evolview (https://www.evolgenius.info/evolview/#/treeview, 9 September 2025) [99,100]. Multiple sequence alignment of ABI4 amino acid sequences was performed using DNAMAN software (version 8.0.8.789, Lynnon Biosoft, San Ramon, CA, USA).

The 2000 bp upstream flanking sequences of the *ABI4* genes were selected as promoter regions and submitted to the PlantCARE online database (http://bioinformatics.psb.ugent.be/webtools/plantcare/html/, 13 September 2025) for in silico analysis of CREs [101].

The secondary structures of BnaABI4 proteins were analyzed via SOPMA (https://npsa.lyon.inserm.fr/cgi-bin/npsa_automat.pl?page=/NPSA/npsa_sopma.html, 15 February 2025) [102,103] and visualized via NetSurfP-3.0 (https://services.healthtech.dtu.dk/services/NetSurfP-3.0/, 16 February 2025) [104]. Tertiary structure models of BnaABI4 proteins were generated via the AlphaFold online server (https://colab.research.google.com/github/sokrypton/ColabFold/blob/main/AlphaFold2.ipynb#scrollTo=11l8k--10q0C, 17 February 2025) [105,106] and subsequently visualized with PyMOL software (version 3.1.3.1, Schrödinger, LLC, New York, NY, USA).

### 4.2. Subcellular Localization

To determine the subcellular localization of *BnaABI4* genes, the four *BnaABI4* genes coding sequence were separately cloned into the P27-GFP vector to generate the BnaABI4-GFP fusion constructs. *Agrobacterium tumefaciens* (GV3101) was used for transient transformation of the 35S::BnaABI4-green fluorescent proteins (BnaABI4-GFP) vector and 35S::GFP (P27-GFP) empty vector by injection into leaf abaxial epidermal cells of 4-5-week-old *Nicotiana benthamiana* [107]. After 36–48 h incubation (25 °C, 16 h light/8 h dark), fluorescence signals were observed using a Leica fluorescence microscope (Leica, Germany). GFP was excited at 488 nm and detected at 500–550 nm, whereas mCherry was excited at 587 nm and detected at 600–650 nm. pBI121-NLS-mCherry was used as the nucleus marker. Experiments were performed with three independent biological replicates, and at least 6 independent tobacco leaf abaxial epidermal cells were analyzed per replicate.

Meanwhile, the BnaABI4-GFP fusion expression vectors were heterologously transformed into *Arabidopsis thaliana*. After successive subculture and screening, genetically stable homozygous transgenic lines were obtained. The primary root tips of 8-day-old homozygous transgenic seedlings were harvested for GFP fluorescence observation under a Leica fluorescence microscope. For each line, root tips from at least ten seedlings were randomly selected for biological replication.

### 4.3. Plant Materials and Growth Conditions

*B. napus* cv. ZS11 was used as WT during this study. For rapeseed regeneration and transformation experiments, ZS11 seeds were surface-sterilised and aseptically sown on half-strength MS medium [108]. All plants were grown in a growth chamber at 25 °C with photosynthetic photon flux density (PPFD) of 150 µmol m^−2^ s^−1^, a 12 h/12 h light/dark photoperiod, and 70% relative humidity.

### 4.4. Vector Construction and Genetic Transformation of B. napus

Total RNA was isolated from seedlings of *B. napus* cv. ZS11 using the Eastep Super Total RNA Extraction kit (Promega, Madison, WI, USA, LS1040) following the manufacturer’s instructions. First-strand cDNA was synthesized using the Thermo Scientific RevertAid First Strand cDNA Synthesis Kit (Fermentas, Waltham, MA, USA, K16225).

We cloned the CDS sequence of *BnaABI4-4*, and subsequently cloned into the pFGC5941 vector using the ClonExpress II One Step Cloning Kit (Vazyme, C112, Nanjing, China). The construct was driven by the CaMV 35S promoter to construct *BnaABI4-4* overexpression lines (OE).

To generate *BnaABI4* knockout mutants (KO) using the CRISPR/Cas9 system, guide RNA (gRNA) targeting *BnaABI4* genes was designed and inserted into the psgR-Cas9-At vector, and the recombinant fragment was subsequently cloned into the pCAMBIA1300 vector [109,110].

All recombinant plasmids verified by Sanger sequencing were transformed into *B. napus* cv. ZS11 via an *Agrobacterium*-mediated genetic transformation system for rapeseed [111,112]. For OE lines, 20 mg/L PPT was used as the selective agent, whereas 20 mg/L hygromycin was adopted for screening KO lines. Positive transgenic lines were first identified by PCR, and further confirmed by Sanger sequencing and RT-qPCR. All primers used in this study are listed in Table S5.

### 4.5. Drought Treatment

WT, OE and four KO lines (KO-1–KO-4) seeds were sown in soil-filled pots for drought stress analysis. All experiments were performed with three biological replicates. All plants were cultivated in a growth chamber at 25 °C under a photosynthetic photon flux density (PPFD) of 150 µmol m^−2^ s^−1^, with a 12 h/12 h light/dark photoperiod, and 70% relative humidity. Drought treatment was initiated when seedlings developed to the 4–5 leaf stage. Well-watered seedlings were then subjected to drought stress for 18 days, followed by a 2-day rewatering recovery. The plants were photographed and observed before and after treatment.

### 4.6. Physiological Measurements

Measurements of Tr and Gs were conducted on the third fully expanded leaf of each plant before drought treatment and during drought stress. A LI-6400XT portable photosynthesis system (LI-COR Biosciences, Lincoln, NE, USA) fitted with a 6400-02B LED red/blue light chamber was used for all determinations. All measurements were taken between 9:00 and 11:00 a.m. The leaf chamber parameters were set as follows: PPFD at 1200 μmol m^−2^ s^−1^, leaf temperature at 25 °C, ambient CO_2_ concentration at 400 μmol mol^−1^, and airflow rate at 500 μmol s^−1^. All values were automatically logged by the instrument.

### 4.7. Stomatal Measurements

To investigate stomatal density, WT, OE and KO-1–KO-4 rapeseed seedlings with uniform growth were selected at the 4–5 leaf stage. The abaxial epidermis was peeled from the third leaf of each seedling while avoiding the main vein, and then prepared into temporary slides. Microscopic observation and image capture were performed at 40X magnification under bright field with a Leica microscope (Leica, Wetzlar, Germany). Stomatal density was quantified by counting the number of stomata in each microscopic field. Thirty biological replicates were conducted in this assay.

### 4.8. Transcriptome Sequencing and Data Analyses

WT and OE plants of *B. napus* cv. ZS11 at the 4–5 leaf stage under normal growth conditions were selected as the sequencing materials. For each genotype, ten individual plants were pooled to form one mixed sample, with three independent biological replicates included per group. Fresh samples were immediately frozen in liquid nitrogen and transported on dry ice to Wuhan Kangce Technology Co., Ltd. (Wuhan, China) for transcriptome sequencing.

Total RNA was extracted from each sample as described above. After quality evaluation, cDNA libraries were constructed and then sequenced on the DNBSEQ-T7 platform (MGI Tech, Shenzhen, China) with paired-end 150 bp (PE150) reads for transcriptome analysis. Raw reads were filtered to eliminate low-quality sequences. The resulting clean reads were mapped to the reference genome of *B. napus* cv. ZS11.v0, which was downloaded from the BnIR database, for subsequent gene expression quantification.

Gene expression levels were normalized to RPKM (Reads per Kilobase per Million Reads) values [113], calculated as: RPKM = total exon reads/(mapped reads (millions) × exon length (KB)). DEGs were identified using the thresholds of |log_2_FC| > 1 and *p* < 0.05.

Functional enrichment analysis of DEGs was performed based on the GO (http://www.geneontology.org, 16 December 2024) [114] and KEGG (http://www.genome.jp/kegg/, 18 December 2024) [115] databases. Bubble plots for GO and KEGG enrichment analyses were generated using GraphPad Prism v9.0, with *p* < 0.05 considered statistically significant for enrichment.

### 4.9. RT-qPCR

Quantitative real-time PCR (RT-qPCR) was performed on a CFX96 Real-Time system (BIO-RAD, Hercules, CA, USA) using Hieff^®^ qPCR SYBR Green Master Mix (No Rox, Cat. No. 11201ES08, Yeasen, Shanghai, China). Total RNA extraction and cDNA synthesis were conducted as described above. *BnaActin7* (*BnaC02G0037200ZS*) was used as the internal control. Gene-specific primers are listed in Table S5. Relative expression levels were calculated using the 2^−ΔΔCt^ method [116].

### 4.10. Statistical Analysis

All the experiments were performed with at least three biological replicates. Data are presented as mean ± standard deviation (SD). Multiple-group comparisons were conducted using one-way analysis of variance (ANOVA) followed by Tukey’s multiple-comparison test. Differences were considered statistically significant at *p* < 0.05. Bars marked with the same lowercase letter indicate no significant difference. Graphs and statistical analyses were performed using GraphPad Prism v9.0.

## 5. Conclusions

In conclusion, the present study systematically identified and functionally characterized four *BnaABI4* paralogs (*BnaABI4-1* to *BnaABI4-4*) in *B. napus* and demonstrated their important roles in stomatal regulation and drought adaptation. Bioinformatic analyses revealed that BnaABI4 proteins belong to the AP2/ERF transcription factor superfamily and contain a highly conserved AP2 domain, while promoter analysis indicated that their expression is potentially regulated by multiple stress- and hormone-responsive signaling pathways.

Functional analyses of WT, OE and KO lines revealed that BnaABI4 acts as a positive regulator of drought tolerance and a negative regulator of stomatal density in *B. napus*. Consistently, overexpression of *BnaABI4-4* reduced Tr, Gs and stomatal density, along with enhanced drought tolerance, whereas KO lines exhibited the opposite phenotypes. Notably, the stronger drought-sensitive phenotype observed in triple- and quadruple-knockout mutants (KO-1, KO-3, KO-4) compared with the double-knockout line (KO-2) suggests that *BnaABI4* paralogs function synergistically during drought responses.

Transcriptome analysis further revealed that BnaABI4 coordinates a complex drought-responsive regulatory network involving ABA signaling, MAPK signaling, ROS homeostasis, cutin and wax biosynthesis, cell wall remodeling, osmotic adjustment, and stomatal development pathways. Key drought-related genes, including *BnaNCED9*, *BnaSnRK2.2*, *BnaRAF43, BnaSPCH*, *BnaSCRM*, *BnaMYC2*, and antioxidant-associated genes, were significantly regulated in OE lines, indicating that BnaABI4 enhances drought tolerance through integrated regulation of stomatal traits, ABA-mediated signaling, and oxidative stress responses. In addition, the observed “low baseline and strong induction” regulatory pattern suggests that BnaABI4 enables drought adaptation while minimizing growth penalties under non-stress conditions.

Collectively, these findings uncover a previously uncharacterized role of ABI4 in regulating stomatal density in *B. napus*, expand current understanding of *ABI4*-mediated drought adaptation in polyploid oilseed crops, and provide valuable candidate genes and theoretical foundations for the molecular breeding and genetic improvement of drought-tolerant rapeseed varieties.

## Data Availability

The data supporting this study can be found in the Supplementary Materials. The raw transcriptome sequencing data generated in this study have been deposited into the Genome Sequence Archive (GSA) of National Genomics Data Center, China National Center for Bioinformation, with accession number CRA043283, accessible at https://ngdc.cncb.ac.cn/gsa (accessed on 3 June 2026) [117,118].

## Supplementary Materials

The following supporting information can be downloaded at: https://www.mdpi.com/article/10.3390/plants15121793/s1, Table S1: Physicochemical properties of plant ABI4 proteins; Table S2: Statistical table of stomatal density in leaves of different *B. napus* genotypes; Table S3: Quality control and mapping information of transcriptome data from WT and OE on *B. napus*; Table S4: The ABI4 protein sequences from different plants; Table S5: Primers used in the thesis.
